# Exploring the Dynamic Interplay Between Foreign Language Enjoyment and Learner Engagement With Regard to EFL Achievement and Absenteeism: A Sequential Mixed Methods Study

**DOI:** 10.3389/fpsyg.2021.766058

**Published:** 2021-10-15

**Authors:** Yunxian Guo

**Affiliations:** School of College English Teaching and Research/Postdoctoral Research Center for Chinese Language and Literature Studies, Henan University, Kaifeng, China

**Keywords:** Foreign language enjoyment, learner engagement, achievement, absenteeism, mixed methods study

## Abstract

Scholarly attention to the feeling of enjoyment experienced in second language acquisition (SLA) has sharply increased in the past 5 years owing to its positive effect on facilitating academic outcomes as well as promoting language learners' well-being. This sequential mixed methods study aims to examine the interplay between Foreign language enjoyment (FLE) and learner engagement (LE) as well as their combined effects on participants' EFL achievement and absenteeism. To this end, we administrated a questionnaire containing the adapted FLE Scale and the four-aspect engagement inventory among 707 Chinese university students and a semi-structured interview among 28 of them. Statistical analysis revealed that FLE was highly and positively correlated with LE, and the causal relationship between the two constructs was reciprocal. Furthermore, both FLE and LE had low correlations with participants' academic achievements, but no significant correlation was found between FLE or LE and absenteeism. However, a higher level of FLE-social was associated with a lower level of absenteeism. Finally, no gender differences were found either in the level FLE or in that of LE. The thematic analysis indicated that FLE was subject substantially to teacher-related variables and the second most significant attractor of FLE was FLE-self. Analysis of the trends of LE indicated that Chinese EFL learners preferred to engage themselves in their English study more emotionally, behaviorally, and cognitively than agentically. Pedagogical implications of the findings for EFL practitioners are also discussed, and suggestions for future research are offered.

## Introduction

The turn of the new millennium has witnessed the modern positive psychology (PP) movement since Martin Seligman became the president of the American Psychological Association. Shifting away from the exclusively pathological orientations toward abnormalities, disorders, and other negative experiences people encounter in general psychology, PP is devoted to “the scientific study of what goes right in life” (Peterson, 2006, p. 4). Focusing on human positive functioning and flourishing at biological, personal, relational, social, institutional, cultural, and global level (Seligman and Csikszentmihalyi, 2000), PP was founded on three pillars: (1) positive experiences, (2) positive character traits, and (3) positive institutions.

To corroborate the practical significance of PP, Fredrickson (2001, 2013) formulated the broaden-and-build theory to further highlight the impact of positive emotions and experiences on nourishing flourishing individuals. The theory states that positive emotions—including joy, interest, contentment, pride, and love, share the ability to “broaden people's momentary thought-action repertoires and build their enduring personal resources, ranging from physical and intellectual resources to social and psychological resources” (Fredrickson, 2001, p. 219). The inherited humanistic concern of PP and the significant progress it has made in social science, coupled with the “social turn” (Block, 2003; Gregg, 2006) and the “individual turn” (MacIntyre, 2014; MacIntyre and Mercer, 2014) of SLA as well as the existing methodological diversity in this avenue, have made the application of PP both desirable and feasible (e.g., MacIntyre, 2014; MacIntyre and Mercer, 2014; Mercer et al., 2018; MacIntyre et al., 2019; Budzińska, 2021; Wang et al., 2021). As a matter of fact, the relatively short marriage of PP and SLA is producing new knowledge in each of the original three pillars (MacIntyre, 2021).

Foreign language enjoyment (FLE), as one of the key positive emotions predicting the performance and well-being of FL learners and a critical factor contributing to the creation of a positive classroom atmosphere, is in alignment with the first and third pillar of PP and has drawn considerable scholarly attention in the past 5 years. A large body of existing literature on FLE has focused on investigating the nature, predictors as well as dynamic features of this positive emotion over a period of time, yet a few recent studies have expanded their research attention to exploring the correlation between FLE and desirable academic outcomes or positive personality traits (e.g., Dincer et al., 2019; Li et al., 2020b; Elahi Shirvan et al., 2021b; Wang et al., 2021). It is worth mentioning that Mercer and Dörnyei (2020) have noticed the association between FLE and LE and pointed out explicitly that FLE has an effect on sustaining learner engagement (LE) despite the fact that the connection between the two constructs has been touched upon implicitly in a couple of previous studies.

Learner engagement (LE) has been one of the hottest research topics in the field of educational psychology (Sinatra et al., 2015) and, with the arrival of PP in second language acquisition (SLA) in the past couple of years, it has been a renewed and burgeoning domain in this avenue due to its core status in successful language learning, maintaining both language teachers and learners' wellbeing and its malleability as well. A majority of existing studies on LE explains its antecedents in light of Self-Determination Theory (SDT) (Ryan and Deci, 2000, 2017) whereas it is worth noting that the satisfaction of the basic psychological needs of autonomy, competence and relatedness which is found to induce LE (Ryan and Deci, 2000, 2017; Noels et al., 2018), to some degree, also facilitates FLE. However, existing research on LE in SLA is relatively sparse (Mercer and Dörnyei, 2020) and few empirical studies have been undertaken so far to explore the dynamic interplay between FLE and LE.

When it comes to absenteeism, there is little doubt that school attendance is highly related to academic achievement as decreased exposure to teachers and teaching would probably reduce the opportunity for learning. Previous studies have found strong significant correlations between attendance and achievement (Roby, 2004) and that high absenteeism predicted dropping out (Rumberger and Lim, 2008). That makes the exploration of factors reducing absenteeism an imperative and meaningful endeavor whereas next to none studies have been undertaken to investigate the effects of FLE and LE on language learners' academic performance and class attendance. To bridge the gap, this study is set to examine the interplay of FLE and LE, and their combined effects on EFL learners' achievement and absenteeism.

## Literature Review

### Foreign Language Enjoyment

Resonating with the advent of PP in SLA, the feeling of enjoyment has been one of the hot topics in recent research on foreign language education. Researchers' interest in the enjoyableness in SLA initiated from their exploration of the possible link between this positive emotion and EFL learners' academic performance. Green (1993) found across 17 activities that enjoyment and perceived learning effectiveness of the tasks did indeed go hand in hand. However, Brantmeier (2005) reported conflicting results from his own data: enjoyment was correlated positively with self-assessed ability as measured by a written recall task, but did not correlate significantly with scores on a multiple-choice test.

Drawing on the insight of PP, Dewaele and MacIntyre (2014) made a giant step toward the study of enjoyment. They explicitly introduced the concept of FLE in SLA and developed the Foreign Language Enjoyment Scale, based on Likert scale ratings of 21 items, which has become the main instrument used to measure FLE. In this paper, they used an internet-based survey to investigate the relationship between FLE and Foreign Language Classroom Anxiety (FLCA), and the statistical analysis revealed that FLE and FLCA were two different dimensions instead of the two sides of a coin, learners experienced a significantly higher level of FLE than FLCA, and there was a modest negative correlation between the two experiences (Dewaele and MacIntyre, 2014). They were also the first researchers who pointed to the gender difference in both emotions, claiming that female participants experience more FLE and FLCA than their male peers. Dewaele and MacIntyre (2016) further advanced the investigation of FLE in the following paper which conducted an in-depth analysis of 2014 research using the aforementioned scale and an attached open question asking participants to give a detailed description of a really enjoyable event or episode in their FL class. Based on the respondents' narratives of their most enjoyable experiences, they contrasted enjoyment with pleasure and conceptualized it as a “complex emotion, capturing interacting dimensions of challenge and perceived ability that reflect the human drive for success in the face of difficult tasks” (Dewaele and MacIntyre, 2016, p. 217). Moreover, they argued that FLE was mediated by both social factors such as a good atmosphere among nice teacher and supportive peers and private factors like learners' sense of pride and success.

Since then, the research on FLE began to flourish and expanded epistemologically, methodologically, and geographically (Mierzwa-Kamińska, 2021), yet majorly following the two research lines that Dewaele and MacIntyre (2014, 2016) have established: (1) investigating the mechanism or contributors of FLE, and (2) examining the dynamics between FLE and FLCA.

In alignment with the first research line, Dewaele et al. (2016) further explored the gender difference in FLE and FLCA at the item level, using both FLE Scale and an open question to collect data. The results, confirming yet refining the findings in the previous study (Dewaele and MacIntyre, 2014), revealed that female participants significantly had more fun and experienced more mild FLCA in the FL classroom. Piechurska-Kuciel (2017) investigated the relationship between learners' command of language and their levels of enjoyment. The results revealed that while reliable social bonds with teacher and peers facilitated FLE, language proficiency was also significantly, positively related to this positive emotion, since better command of language was usually connected with greater control perception and proficient learners were more likely to benefit from the recognition of the value of language proficiency. Elahi Shirvan and Talebzadeh (2017) used an idiodynamic method to discern the effect of different conversational topics on the dynamics of FLE by recording rapid moment-to-moment changes of this positive experience. Results from this research indicated that FLE was a dynamic system varied both inter-personally and intra-personally and the topic was an attractor state for learners' enjoyment. These findings suggested that teachers could increase language learners' level of enjoyment with the selection of proper conversational topics and the control of the extent of their difficulty and amusement in classroom interactions. Dewaele et al. (2018) investigated whether, and to what extent, FLE and FLCA were connected with learner-internal and teacher-specific variables. The results indicated that FLE and FLCA were subject to both learner-internal and classroom-specific factors. To elaborate, older, more experienced, more proficient students experienced higher levels FLE. What's more, enthusiasm toward the FL and the FL teacher, a lot of FL use of the target language by the teacher in class, a big proportion of time students spent on speaking were all reported to contribute to higher levels of FLE. Teachers were found to play a more vital role in boosting students' FLE than alleviating their FLCA. De Smet et al. (2018) examined how target language influenced FL learner's FLE and FLCA. They found that bilinguals had higher levels of FLE and lover levels of FLCA than their monolingual counterparts. In addition, target language played a fundamental role in classroom emotional engagement. For example, English learners reported significantly more FLE and less FLCA and than Dutch learners. Talebzadeh et al. (2019) explored the mechanisms and dynamics of enjoyment contagion in a course of foreign language. The findings indicated that automatic mimicry was the main mechanism of enjoyment contagion in teacher-student interactions. And this was shaped by the application of facial expressions, gestures, and postures like laughter, vocalic expressions, smiling, nodding, and leaning forward.

By the same token, Moskowitz and Dewaele (2020) administered an online survey on 163 adult Spanish EFL learners to explore possible links between FL learners' intellectual humility (IH) and FLE and FLCA. The results revealed that IH had a mixed and complex relationship with FLE and FLCA, with some IH domains negatively predicting FLE and both positively and negatively predicting FLCA. Ahmadi-Azad et al. (2020) investigated the role of Big Five personality traits of EFL teachers in facilitating learners' FLE and found that teachers' openness, extroversion, and agreeableness were significantly, positively associated with learners' FLE while their conscientiousness and neuroticism did not have significantly similar effects.

In line with the second denomination, Dewaele and Dewaele (2017) investigated how FLE and FLCA changed over time among secondary students, basing on a pseudo-longitudinal design. Statistical analysis revealed that while the weak negative correlation between FLE and FLCA remained stable, the variables influencing positive and negative emotions did change over time. For example, the effect of the teacher grew over time on FLE but not on FLCA. Set in the Iranain context, Elahi Shirvan and Taherian (2018) conducted a longitudinal study, using the latent growth curve modeling (LGCM), to investigate the growth and changing trends of university students' FLE and FLCA. Statistical analysis revealed that the growth of FLE and FLCA were strongly, negatively correlated though their initial states were weakly associated. What's more, the initial states of either FLE or FLCA did not predict their growth through the semester. In addition, the growth of both the positive and negative emotions were subject to both inter-individual and intra-individual variables. Moreover, Elahi Shirvan and Talebzadeh (2020) used the retrodictive qualitative modeling (RQM) to explore the signature dynamics of FLE and FLCA. The study found that the prototype contributors of those emotions were the influence of the teacher, personal goals, a perfectionist image of oneself and dissatisfactory and unsuccessful experiences in the past. Dewaele and Dewaele (2020) investigated whether FL learners experienced similar levels of FLE and FLCA in the same language if they had two different teachers at a single point. Statistical analysis revealed that students had significantly higher level of FLE with the main teacher while their FLCA level was stable with both teachers, suggesting that teachers' creation of a positive emotional atmosphere in class contributed to the higher FLE score. More recently, Elahi Shirvan et al. (2021a) adopted a longitudinal confirmatory factor analysis-curve of factors model (LCFA-CFM) approach to explore the temporal growth of FLE over time and how it evolved through the L2 course (FLE) among adult EFL learners. The results revealed that learners with lower initial FLE experienced a faster increase in FLE over time, which can be can be attributed to learner's motivation, changing attitude to L2 learning and the supportive role of the teacher. Elahi Shirvan et al. (2021b) used a factor of curves model (FCM) to further trace the longitudinal co-development of adult EFL learners' private-FLE and social-FLE. The results indicated that there was a significant increase over time in both subdomains and the increase could be largely explained by the global factor of FLE.

Apart from the aforementioned two denominations, there were also studies undertaken to investigate the possible links between FLE and desirable academic outcomes or other positive personality traits. Dewaele and Alfawzan (2018), for instance, investigated the effect of FLE and FLCA on learners' FL performance. Statistical analysis from their study showed that there was a significant, positive relationship between FLE and students' self-reported test results and a slightly bigger one than the significant, negative relationship between FLCA and their achievement. This study also suggested that perception of the FL teacher and teachers' pedagogical practices played a crucial role in facilitating FLE. In another study, Dewaele and Dewaele (2018) explored the interplay of learner-internal and learner-external variables to predict students' Willingness to Communicate (WTC) and found that high level of FLE was positively associated with learners' WTC in FL classroom. In another study undertaken by Elahi Shirvan et al. (2021b), a bivariate latent growth curve model (LGCM) was used to investigate the growth of FLE and L2 grit over time. The findings indicated an increasing trend in the association between the growth levels of both variables and an increase in the level of FLE among the participants was strongly correlated with an increase in the level of L2 grit during the whole course.

Different from previous studies concentrating on EFL learners' feeling of enjoyment, Mierzwa (2019) made a pioneering investigation of the level of FLE among FL teachers in Poland to examine the possible sources of the positive feeling. The results revealed teachers experienced a relatively high level of enjoyment both in FL learning and FL teaching. And, female teachers scored significantly higher in FL learning enjoyment than their male counterparts while there was no gender difference in FL teaching enjoyment. In addition, the study also found that FLE was more related to learner-internal and teacher-specific variables than to the behavior of peers and the atmosphere in the classroom, corroborating the findings of Li et al. (2018).

Inspired by the scholarly attention to FLE in the Western world, Chinese scholars began their exploration of FLE in Chinese educational and cultural context, following practically similar research course of studies in other parts of the world. Li et al. (2018) initiated their exploration of FLE among Chinese EFL learners by capturing the uniqueness of Chinese psychometric properties. Using the Chinese version of FLE scale they devised, Li et al. (2018) examined the factors facilitating FLE in Chinese EFL context and the results revealed that Chinese EFL learners' enjoyment was formulated by the dimensions of FLE-teacher, FLE-private and FLE-atmosphere among which EFL instructors played a crucial role in creating a positive classroom. Jiang and Dewaele (2019) examined to what extent Chinese undergraduate EFL learners' FLE and FLCA were different from that of learners outside China. Statistical analysis illustrated that Chinese EFL learners experienced similar levels of FLE but higher levels of FLCA compared to the international sample collected by Dewaele and MacIntyre (2014). Their study further confirmed the previous findings that FLE was more strongly predicted by teacher-specific variables while FLCA was mostly subject to learner-internal variables except that Chinese learners disliked teachers' unpredictable behaviors. Li et al. (2020b) administered questionnaires among Chinese secondary and university students respectively to examine the combined effects of Trait Emotional Intelligence (TEI) and Classroom Environment (CE) on FLE and FLCA. The results of both samples indicated that TEI and CE had both independent and joint effects on FLE and FLCA.

Despite the fact that a vast majority of extant literature on FLE focused on the conceptualization, measurement, and antecedents of this positive emotion, there emerged studies which correlated FLE with the language learning process. Several Chinese studies focused their research attention to the mediating effects of FLE on learners' academic outcomes. Li (2019) examined the complex relationships between Chinese EFL learners' trait emotional intelligence (TEI), FLE, and achievement. The results revealed that there were small to medium correlations between students' TEI, FLE, self-perceived English performance, and their actual English achievement. Moreover, FLE played some yet indirect part in mediating learners' TEI to influence their self-assessed achievement and actual scores. Wei et al. (2019) investigated the mediating role of FLE and classroom environment (CE) in the relationship between Chinese EFL learners' grit and performance. The results revealed that students' grit was positively associated with their performance and that FLE mediated the relationship between their grit and performance. In addition, Positive CE played a part in increasing the impact of grit on both FLE and achievement. Their research also indicated some gender differences with females reporting higher scores in grit, FLE, CE, and performance than their male peers. Li et al. (2020a) examined the interplay between Chinese EFL learners' FLE and FLCA and found that FLE was positively related to their self-rated proficiency while FLCA was significantly, negatively related to that. The latest research concerning FLE was conducted by Zhang et al. (2021) who investigated whether Thai EFL learners' FLE and English proficiency influenced their preference for Written Corrective Feedback (WCF). The results showed that learners preferred more explicit types of WCF irrespective of their language proficiency and FLE level. However, the FLE level seemed positively linked to their perception of the value of WCF in terms of scope. Other studies further found that FLE could lead to better academic achievement (Jin and Zhang, 2018; Li et al., 2020a), increase their engagement in the language learning process (Jin and Zhang, 2019) argued, or boost social-behavioral learning engagement (Dewaele and Li, 2020).

### Learner Engagement

Engagement, as an antidote to signs of student alienation such as classroom boredom and absenteeism and a booster for academic achievement, has been one of the hottest research topics in the field of educational psychology (Sinatra et al., 2015). For this reason, it has been brought under investigation across multiple contexts and subject matters (e.g., Fredricks et al., 2004; Christenson et al., 2012; Fredricks and McColskey, 2012; Lawson and Lawson, 2013). Engagement is also one of the pivotal occupations and cornerstones of positive psychology (PP), constituting the E construct of PERMA framework to promote individual wellbeing (Seligman, 2011). Its crucial role in both fields has made engagement a fledgling darling in SLA in the past few years (see Pekrun and Linnenbrink-Garcia, 2012; Philp and Duchesne, 2016; Mercer, 2019; Mercer and Dörnyei, 2020; Mystkowska-Wiertelak, 2020) since learner engagement lies at the heart of successful language learning and is one of the predictors of learners' happiness. Moreover, a growing body of research suggests that learner engagement is malleable and subjects to deliberate interventions and specific teacher behaviors (Harbour et al., 2015). These reasons make language learner engagement an interesting and meaningful domain to explore in depth whereas existing research on engagement in SLA is relatively sparse (Mercer and Dörnyei, 2020).

It has been widely accepted that engagement is a multidimensional construct which makes the understanding and definition of it quite a challenge. Fredricks et al. (2004) defined engagement as a notion comprising three core components: behavioral, emotional, and cognitive dimensions. Yet the endeavor in conceptualizing and theorizing this construct never stops owing to its complexity. Later, Svalberg (2009) as well as Philp and Duchesne (2016) brought forth “social” as the fourth component, arguing that cognitive, behavioral, social, and emotional dimensions operated interdependently and mutually influenced one another. However, the “social” dimension aroused skepticism since all the other components have been socially situated (Reeve, 2012; Mercer and Dörnyei, 2020). To further conceptualize and theorize learner engagement, Reeve and Tseng (2011) and Reeve (2013) added an alternative “agentic engagement” as the fourth facet of this construct. Agentic engagement was defined as “students' constructive contribution into the flow of the instruction they receive” (Reeve and Tseng, 2011, p. 258) and considered as a “proactive, intentional, collaborative, and constructive student-initiated pathway to greater achievement” (Reeve, 2013, p. 579).

All of the aforementioned conceptualization and theorizing of engagement lay emphasis on the behavioral involvement in various level ecologies of engagement, or share the reference to action (Mercer and Dörnyei, 2020; Mystkowska-Wiertelak, 2020). This resonates with Skinner et al.'s (2009, p. 225) description of engagement as “energized, directed, and sustained actions.” It is this actional or behavioral dimension that differentiates engagement from another affinitive notion—motivation (Oga-Baldwin and Nakata, 2017; Mercer, 2019). As a matter of fact, studies of engagement to some extent initiated from the more conventional notion: motivation. Prevailing views include seeing engagement as a descriptor of motivation (Philp and Duchesne, 2016) or an observable manifestation of cognitive and emotional activity in the form of participation and enjoyment (Reeve, 2012), and regarding motivation as the hidden mental reality encompassing conscious and unconscious drives (Reeve, 2012), the precursor (Pekrun and Linnenbrink-Garcia, 2012), or antecedent (Christenson et al., 2012) of engagement, to name just a few. Reeve and Lee (2014) also tested the impact that changing students' classroom engagement had on their longitudinal classroom motivation. Results revealed that high-quality classroom engagement facilitated students' in-course motivation, especially their psychological need satisfaction and self-efficacy. A similar study undertaken by Oga-Baldwin and Nakata (2017) suggested that engagement strongly predicted intrinsic motives but negatively predicted extrinsic motives. Male students were found to have lower engagement, lower internally regulated motives, and higher externally regulated motives.

Svalberg (2009, 2017) was among the first researchers who explicitly brought up the topic of engagement in SLA and laid a sound theoretical basis for the study of engagement in language learning. She advanced the term “engagement with language (EWL)” and defined it as “a cognitive, and/or affective, and/or social state and a process in which the learner is the agent and the language is the object and may be the vehicle (means of communication)” (Svalberg, 2009, p. 244). To resolve terminological and methodological confusion, she also made a clear distinction between engagement and neighboring terms of involvement, commitment, and motivation, arguing that each of the three concepts covered only part of the nature of engagement yet failing to capture the entirety of the construct (Svalberg, 2009). In another paper, Svalberg (2017) conducted a diachronic delineation of “engagement” in the literature to better understand its role in language awareness and language learning, and to situate this construct in relation to other similar notions like contextual engagement, task engagement, and engagement with corrective feedback. It was found that meaningfulness, which is linguistic, social, or individual in nature and predicted by purposefulness, utility, and enjoyment, is a highly influential factor for engagement research.

Across a bulk of previous studies on engagement, Self-Determination Theory (SDT) (Ryan and Deci, 2000, 2017) was the most frequently adopted theoretical framework to explain the antecedents of learner engagement. The basic assumption of SDT approach is that greater engagement can be predicted when basic psychological needs for autonomy, competence and relatedness are met (Ryan and Deci, 2000, 2017; Noels et al., 2018). Autonomy refers to learners' need to exercise agency in shaping their own learning according to their beliefs, values, and interests (Ryan and Deci, 2017). Competence concerns learners' deep-held belief that they can face up to challenges and bring their action to the desired end. Learners' sense of relatedness is more context-dependent, relying on the feeling of belonging provided by supportive teachers and peers (Jang et al., 2012; Ryan and Deci, 2017; Dincer et al., 2019).

To better understand the predictors of engagement, several researches were conducted in the light of the SDT. For example, Skinner et al. (2008) used a motivational development model to investigate the internal dynamics of four indicators of behavioral and emotional engagement and disaffection and how teacher support and students' self-perception of their competence, autonomy, and relatedness effected changes in these indicators over the school year. The study found that emotional engagement positively predicted changes in behavioral engagement. Furthermore, teacher support and students' self-perceptions had a similar effect when it comes to increasing behavioral engagement and decreasing disaffection. Noels (2009) examined the mediating role of enhanced engagement, predicted by the satisfaction of psychological needs of autonomy, competence, and relatedness, in supporting learner motivation. Furthermore, a sense of autonomy was argued to be the strongest facilitator for volitional engagement in the learning process since it conferred learners a feeling of identification with ethnolinguistic groups.

Noels et al. (2018) conducted a longitudinal investigation of the causal claim that learners tend to be more engaged and motivated when they feel autonomous, competent, and related to others in their learning environment. Statistical analysis confirmed an early argument that engagement arose through self-processes, particularly a self-determined motivational orientation (Ryan and Deci, 2017), and further revealed that earlier engagement levels enhanced later motivational orientations and that earlier motivational levels had an effect on learners' later perceptions of psychological need fulfillment and later engagement. Moreover, Mercer (2019) discussed antecedents of engagement through the lens of SDT and argued that students' feeling of competence, autonomy, and relatedness prepared them for fuller engagement and necessary actions taken in the language learning process. By the same token, Dincer et al. (2019) examined the relations between EFL classroom context, self, engagement, and academic outcome and found that engagement was predicted when students' psychological needs were met. They also found that higher emotional and agentic engagement was positively associated with academic achievement while cognitive engagement predicting decreased absenteeism.

When exploring the antecedents of engagement, several researchers focused their attention on the dynamics between classroom context and EFL learners' engagement. Ryan and Patrick (2001) examined the dynamic relation of students' perceptions of the social environment (classroom) to their motivation and engagement and found that higher-order classroom social environments were strong predictors of changes in learners' motivation and engagement. And students' perceptions of teacher support, and the teacher as promoting interaction and mutual respect facilitated their motivation and engagement whereas their perceptions of the teacher as promoting performance goals led to negative changes in motivation and engagement. The relation between teacher-student interaction and engagement also aroused the interest of Reeve et al. (2004) who tested whether classroom teachers could incorporate autonomy support into their motivating styles as a way to promote their students' engagement during instruction. The results showed that trained teachers were significantly more autonomy-supportive behaviors, and more importantly, teachers autonomy support was significantly, positively related to students' engagement. Reeve (2012) drew on the insight of SDT to explain how classroom conditions acted as variables to either support or neglect and frustrate students' motivation, engagement, and positive classroom functioning. The study further identified the interactive relation between engagement and the learning environment. Qualitative analysis of another study (Dincer et al., 2019) also indicated that a positive social atmosphere where teachers' autonomy-support was perceived played a crucial role in supporting students' engagement. Teachers' role in facilitating learner engagement was also examined by Mystkowska-Wiertelak (2020) who found that teachers tended to concentrate on the improvement of students' behavioral engagement and had little access to the emotional dimension and less concern for cognitive or social components of the construct. A holistic investigation of learner engagement drawing on insights from PP was conducted by Mercer and Dörnyei (2020). They examined how teachers could develop students' positive emotions in order to promote their active engagement (Liu, 2021), pointing out that feeling of enjoyment learners derive from teacher-student rapport, quality peer relations, and performing tasks with balanced challenges etc., was one of the key elements to sustain their engagement (Mercer and Dörnyei, 2020). The association between FLE and LE was also identified by Dewaele and Li (2020). Moreover, learner engagement was found to be positively related to other teacher-student interpersonal communication behaviors (Xie and Derakhshan, 2021), such as teacher care, rapport (Derakhshan et al., 2021), nonverbal immediacy, and credibility behaviors (Derakhshan, 2021).

The current empirical study is warranted due to several research gaps in the existing literature. First and foremost, there is still a scarcity of empirical studies investigating the effect of FLE on factors vital for successful FL learning, notably learner engagement, despite that FLE is no longer an underestimated emotion (Wang et al., 2021) and a number of studies have expanded our insights into the nature as well as the mechanism of FLE. Second, although FLE has been pinpointed as an significant antecedent of LE in recent research, no empirical study have been carried out to examine the interplay of the two constructs. Finally, no empirical study has been conducted to explore the associations among FLE, LE, achievement and absenteeism. To fill these gaps, this study aims at investigating the impact of FLE on LE, their interplay, and their combined effects on participants' EFL achievement and absenteeism.

## Methods

### Participants

Respondents for the questionnaire in this present study were 707 non-English major undergraduates from three comprehensive universities in central China (273 males, 405 females, and 29 who preferred not to specify their gender). The average age of these participants was 19.4 years old (*SD* = 2.570). Most of them were first-year students (64.2%) and sophomores (14.9%) for whom English was a compulsory course. They were required to take two general English courses, *English Listening and Speaking* and *English Reading and Writing*, respectively, for 2 h per week and for four consecutive semesters. A small proportion of the participants were junior (11.3%) and senior students (9.6%) who were still learning English to sit various tests such as CET (College English Test) band 6, national post-graduate entrance examination, IELTS and TOEFL.

Participants for the semi-structured interview were 28 freshmen from the 707 respondents (13 males, 15 females, and 1 who preferred not to specify the gender). Their average age was 19 years old (*SD* 0.881).

### Instruments

The present study collected both quantitative and qualitative data *via* a questionnaire and a semi-structured interview, respectively. Both data were obtained *via* the electronic versions which were distributed through WeChat (a Chinese multi-purpose messaging app). The questionnaire started with obtaining participants' consent followed by a section collecting their demographic information including gender, age, and level of education. The ensuing section contained two measures: the FLE scale and the learner engagement scale. The last section of the questionnaire asked participants to report their achievements as well as their absenteeism. Students rated their English exam score with a 4-point scale from low (0–59) to high (90–100) following the university grading system (0–59 = 1, 60–69 = 2, 70–89 = 3, 90–100 = 4) (Junior and senior undergraduates reported their self-perceived English proficiency.). Higher scores indicated higher academic achievements. And they also self-reported their course attendance throughout that term using a 4-point scale ranging from no class absences (0) to many class absences (≥5) (0 = 1, 1–2 = 2, 3–4 = 3, ≥5 = 4). Higher scores indicated greater absenteeism. Students who missed 5 or more classes would automatically fail the course.

The qualitative phase of the study involved a semi-structured interview containing 15 open-ended questions regarding classroom atmosphere, psychological needs, classroom engagement, self-perceived performance, and suggestions for improving the course.

### Foreign Language Enjoyment Scale

An adapted and bilingual version of FLE scale including 17 items extracted from the original one developed by Dewaele and MacIntyre (2014, 2016) was used to measure participants' FLE. The first nine items reflected the private dimension of FLE and the following eight items indicated its social dimension. All items were positively phrased and juxtaposed with Chinese translations in brackets. Participants were asked to indicate to what extent they agreed with each item on a standard 5-point Likert scale (strongly disagree = 1, disagree = 2, No idea = 3, agree = 4, and strongly agree =5). Internal consistency of the 17 items was assessed, using Cronbach's Alpha, and the results indicated that the FLE scale used in the present study showed a very high internal reliability (*Alpha* = 0.936).

### Learner Engagement Scale

We used the bilingual version of the learner engagement scale redevised by Reeve and Tseng (2011) which consisted of 22 items assessing four aspects of learner engagement: agentic, behavioral, emotional, and cognitive. Corresponding Chinese translations of all items were provided in brackets. All items were rated on a 5-point Likert scale (Never = 1, Rarely = 2, Sometimes = 3, Often = 4, and Almost always = 5).

The five-item *agentic engagement* measure was originally developed by Reeve and Tseng (2011). Miserandino's (1996) five-item task involvement questionnaire was used to assess *behavioral engagement*. For the assessment of *emotional engagement*, four positively-valenced items reflecting energized emotional states (i.e., enjoyment, interest, curiosity, and fun) were extracted from Wellborn's (1991) conceptualization of students' emotional engagement. *Cognitive engagement* was assessed using Wolters (2004) briefer version of the learning strategies questionnaire containing eight items. The four measures in the present study showed high internal reliability (*Alpha* = 0.89).

### Semi-Structured Interview

Semi-structured interviews were conducted with 28 participants. After giving consent and providing demographic information including their gender, age, and level of education, participants were asked to answer 15 open-ended bilingual questions either in Chinese or in English to make sure they expressed their ideas clearly and accurately. The interview questions (see the English version of the semi-structured interview in the Appendix) were primarily designed to learn about (1) the sources of participants' FLE *via* their rating of the reasons for which they enjoy leaning English, description of enjoyable episodes in English class, comments on the English course, the teacher and the classroom atmosphere, and (2) how they were engaged in English learning in the aspects of agentic, behavioral, emotional, and cognitive.

### Procedure and Data Analysis

The present study was operationalized in two stages: the questionnaire phase and the semi-structured interview phase. In the first stage, questionnaires were distributed and collected online through a Chinese multi-purpose messaging app, WeChat and 707 samples were obtained. In the second stage, 28 participants who agreed to further engage in the present study were asked to answer the interview questions and submit the questionnaires on the same online platform.

Accordingly, data analyses of the study proceeded in two phases. Descriptive analyses, independent-samples *T*-test, correlation analyses, and regression analyses were conducted using SPSS 23.0 in the first phase to have a panoramic view of Chinese EFL learners' FLE and engagement, and more importantly, to identify the relations between FLE and learner engagement and their single and combined effects on achievement and absenteeism. In the second phase, qualitative data were analyzed with Nvivo 12 Plus to elicit themes facilitating participants' FLE and indicating their engagement.

## Quantitictive Results

### Levels of FLE and Learner Engagement

Average scores on the 5-point scale were calculated for FLE (*M* = 3.82, *SD* = 0.65) and separately for FLE-private (*M* = 3.63, *SD* = 0.74) and FLE-social (*M* = 4.03, *SD* = 0.70). Means of each of the two aspects were compared and the results displayed in Table 1 revealed that participants scored significantly higher in FLE-social than in FLE-private.

**Table 1:** Means comparison of different aspects of FLE and LE.

	***F***	***P***
FLE-private * FLE-social	25.05	0.000^***^
Agentic LE * Behavioral LE	28.32	0.000^***^
Agentic LE * Emotional LE	28.11	0.000^***^
Agentic LE * Cognitive LE	16.97	0.000^***^

*** *p < 0.0001; (all two-tailed tests)*.

Average scores on the 5-point scale were calculated for LE (*M* = 3.54, *SD* = 0.71) and separately for agentic LE (*M* = 2.90, *SD* = 1.0), behavioral LE (*M* = 3.77, *SD* = 0.75), emotional LE (*M* = 3.79, *SD* = 0.80) and cognitive LE (*M* = 3.67, *SD* = 0.75). Means of each of the four aspects were compared and the results displayed in Table 1 showed that participants reported significantly lower scores in agentic LE than in behavioral LE (*f* = 28.32, *p* < 0.001), emotional LE (*f* = 28.11, *p* < 0.001) or cognitive LE (*f* = 16.97, *p* < 0.001).

### Gender Differences in Levels of FLE and Learner Engagement

Independent-samples *t*-tests revealed that there were no significant gender differences for either FLE (*df* = 469, *t* = 1.15, and *p* = 0.25) (Females FLE *M* = 3.80, *SD* = 0.56; Males FLE *M* = 3.86, *SD* = 0.75) or LE (*df* = 500, *t* = 1.38, and *p* = 0.17; Females LE *M* = 3.51, *SD* = 0.65, Males LE *M* = 3.59, *SD* = 0.80).

### Interplay Between FLE and Learner Engagement

First, we conducted a series of Pearson correlation analyses to investigate the correlations between FLE and LE. The results summarized in Table 2 showed that FLE was significantly highly and positively correlated with LE. To elaborate, FLE-private and FLE-social had, respectively, high and moderate correlations with LE.

**Table 2:** Correlations between FLE and LE.

	**LE**	
	***r***	***p***
FLE	0.784	0.000^***^
FLE-private	0.753	0.000^***^
FLE-social	0.639	0.000^***^

*** *p < 0.0001 (all two-tailed tests)*.

Considering the significant correlation results between FLE and LE, we performed multiple linear regression analyses, taking FLE and LE as well as their different aspects as predictor variables, respectively, to explore the interplay of the two constructs. FLE and its both aspects were entered into the regression model, respectively for LE as the predicted variable. Significant regression equation models were found (*F* = 1,124.791, *p* < 0.0001, and Adjusted *R*^2^ = 0.614; *F* = 573.814, *p* < 0.0001, and Adjusted *R*^2^ =0.619). According to the regression model summarized in Table 3, 61.4% of the variance of LE was explained by FLE, without any clear evidence of multicollinearity [VIF (1.000) <3]. Comparatively speaking, FLE-private displayed a better predictive power (*B* = 0.538) on LE than FLE-social (*B* = 0.304). LE and its four aspects were entered into the regression model, respectively, for FLE as the predicted variable. Significant regression equation models were found (*F* = 1,124.791, *p* < 0.0001, Adjusted *R*^2^ = 0.614; *F* = 291.557, *p* < 0.0001, Adjusted *R*^2^ = 0.622). In contrast, emotional engagement had a better predictive power (*B* = 0.259) on FLE than agentic (*B* = 0.140), behavioral (*B* = 0.137), or cognitive engagement (*B* = 0.187).

**Table 3:** Results of multiple regression analyses.

**Predictor variables**	**Adjusted *R*^**2**^**	***B***	***t***	***p***	**VIF**
FLE	0.614	0.851	33.538	<0.0001	1.000
FLE-private	0.619	0.538	18.564	<0.0001	1.689
FLE-social	0.619	0.304	9.953	<0.0001	1.689
LE	0.614	0.304	33.538	<0.0001	1.000
Agentic LE	0.622	0.140	7.218	<0.0001	1.703
Behavioral LE	0.622	0.137	7.181	<0.0001	3.818
Emotional LE	0.622	0.259	3.500	<0.0001	3.672
Cognitive LE	0.622	0.187	6.060	<0.0001	2.353

### Interactions Between FLE, LE, and Academic Achievement and Absenteeism

A series of correlation analyses were conducted to explore the combined effects of FLE and LE on learners' achievement and absenteeism. The results (see Table 4) revealed that both FLE (including FLE-private and FLE-social) and LE had significant yet low, positive correlation with learner achievement (FLE *r* = 0.220, *p* < 0.0001; LE *r* = *0*.217, *p* < 0.0001). Moreover, FLE-social was significantly, negatively correlated with learner absenteeism (*r* = −0.108, *p* < 0.05) while none of FLE, FLE-private or LE demonstrated significant correlations with it.

**Table 4:** Correlations between FLE, FLE-private, FLE-social, and LE and achievement and absenteeism.

	**Achievement**		**Absenteeism**	
	***r***	***p***	***r***	***p***
FLE	0.220	0.000^***^	−0.069	0.067
FLE-private	0.272	0.000^***^	−0.023	0.534
FLE-social	0.109	0.004^**^	−0.108	0.004^**^
LE	0.217	0.000^***^	−0.045	0.233

*** *p < 0.0001*;

** *p < 0.01 (all two-tailed tests)*.

## Qualitative Results

### Participants' Description of Sources of FLE

Nvivo 12 Plus was used to conduct thematic analysis of the participants' answers to interview questions, given the fact that employing a “Computer-Assisted Qualitative Data Analysis Software” (CAQDAS) will remarkably enhance the credibility of the coding process (Baralt, 2012). The 28 interviewees were numbered, and their responses were compiled in single word files prior to the coding process. Diverse themes of FLE emerged from participants' descriptions of the most enjoyable episodes in English class, listing of reasons they enjoy learning English and comments on the English course, the teacher, and the classroom atmosphere. In the open coding phase, the analyst read the transcribed data and generated some initial codes. Then, in axial coding phase, the initial codes was compared and grouped under higher-order headings. Finally, in the selective coding phase, the generated themes were categorized into three main themes, *FLE-self* (Participants' *per se* were the primary source of FLE), *FLE-teacher* (Teacher and teacher-related elements were attractors of FLE), and *FLE-peer* (Peer behaviors or peer interaction were the antecedents of FLE), with reference to the coding approaches of Dewaele and MacIntyre (2014) and Jiang and Dewaele (2019). Descriptions touching upon multiple themes had to be categorized differently. Thus, there might be some overlapping coding owing to the complexity of thematic categorization. Table 5 summarizes the sources of FLE emerging from the qualitative data and the number of tokens under each category and subcategory and in total.

**Table 5:** Three Categories of FLE and the number of tokens in the feedback of 28 interviewees.

**Category**	**FLE-teacher**	**FLE-self**	**FLE-peer**
Source (the number of takens)	Stimulating classroom activities (27), positive classroom atmosphere (25), teacher-student rapport (22), teaching skills (19), positive teacher character traits (14), teacher credibility (7), and teaching style (6)	Pursuit of novel knowledge (26), utilitarian needs for English proficiency tests (23), pride of mastering a FL (19), interest (11), and good language performance (8)	Peer support (7), meeting new friends (3)
In total	87	123	10

As is shown in Table 5, participants' FLE is mostly related to teacher-external variables followed by categories of FLE-self and FLE-peer. Among FLE-teacher, stimulating classroom activities, positive classroom atmosphere, and teacher-student rapport are the most frequently mentioned sources of enjoyment. Diversified classroom activities with appropriate challenges, related to students' immediate concern, allowing them chances to present themselves and enhancing interaction, such as debating, translation competition, and text presentation, were reported as most conducive to their FLE. Among the category of FLE-self, participants mentioned that acquiring novel knowledge such as syntactic rules, new vocabulary, and western culture and customs contributed the most to their feeling of enjoyment. Utilitarian needs to get a good grade in multiple English proficiency tests to pass the final exams, CET (College English Test) band 4 or band 6, obtain a bachelor degree, get enrolled to graduate schools home and abroad were also mentioned as an influential attractor to participants' sustained FLE. Among the category of FLE-peer, peer interaction was mentioned as an important factor for participants' FLE.

### Participants' Description of Trends of LE

The codification went through the same stages with that of FLE. Diverse themes emerged from participants' descriptions of their feelings of the English course, their performances in class, the learning strategies they deployed, whether and how they ask questions or express preferences and opinions, and their attendance were first brought into initial codes in the opening coding phrase and further classified into higher-order headings in the axial phase. Finally, in the phase of selective coding, previously generated codes were categorized into the four main themes of the emotional, behavioral, cognitive and agentic dimensions of LE. Those descriptions involved multiple themes were categorized differently. Thus, there might be some overlapping coding owing to the complexity of thematic categorization. The results were summarized in Table 6.

**Table 6:** Trends of LE and the number of tokens in the feedback of 28 interviewees.

**Category**	**Agentic LE**	**Behavioral LE**	**Emotional LE**	**Cognitive LE**
Trends (the number of tokens)	Communicating opinions and preferences after class (17), asking questions in class occasionally (15) communicating opinions and preferences after class in class (6), asking for clarification in class (3)	Engaging in classroom interactions (25), attending class regularly (21), listening to the teacher attentively (19), taking notes (16), presenting in class (9)	Enjoying engaging in class activities (27), enjoying harmonious teacher–student and peer relationships (27), enjoying the class atmosphere (25), interested in English (11), enjoying teamwork with peers (3)	Internalizing knowledge through repeated practice (19) resorting to internet for further information (16), previewing and identifying problems before class (13), reciting (7), and constant accumulation (6)
In total	41	90	93	61

Table 6 shows that stimulating classroom activities, harmonious teacher-student and peer relationships and a positive classroom atmosphere are pivotal factors inducing participants' deep emotional engagement. Taking part in class interaction, attending the class regularly, listening attentively, and taking notes assiduously were mentioned as the most prevalent ways participants engaged in English learning behaviorally. For cognitive engagement, the repeated practice was mentioned as the most important way participants adopted to consolidate and internalize linguistic knowledge. Going to the internet for further course-related information was reported as another influential way they used for cognition. Participants did not report an active agentic engagement and preferred to raise questions for clarification in class very occasionally, leaving their opinions and comments conveyed to the teachers after class.

## Discussion

We first examined the levels of FLE and LE of Chinese EFL learners. Participants reported an average of 3.82 of FLE which is equal to the mean (3.82) reported by the international sample of Dewaele and MacIntyre (2014). This suggests that Chinese EFL learners are having fun in learning English, probably due to a weakening emphasis on examination-oriented education in Chinese education policy in recent years. To elaborate, participants reported significantly stronger FLE-social than FLE-private, which confirms previous findings that FLE subjects more to learner-external factors, largely teacher-related variables (Dewaele and Dewaele, 2017, 2020; Dewaele et al., 2018; Elahi Shirvan and Taherian, 2018; Jiang and Dewaele, 2019; Ahmadi-Azad et al., 2020). This makes it significant and imperative for EFL teachers to perceive the potential affordances of FLE and actualize them as utilized and shaped ones by improving their agency capacity (Elahi Shirvan and Taherian, 2020) or teacher-student interpersonal skills (Xie and Derakhshan, 2021).

Participants reported an average of 3.54 of overall learner engagement, yet a significantly lower level of agentic engagement, indicating that Chinese EFL learners tended to be more emotionally, behaviorally, and cognitively engaged with language learning than agentically. We conjecture that this could be attributed to the tradition of “honoring the teacher and respecting his teaching” in the Chinese educational context where certain agentic behaviors such as recommending a goal or objective to be pursued or communicating likes and dislikes freely, if not performed properly, might be regarded as being impolite and a compromise on the teacher's prestige and dignity.

Secondly, the present study examined the possible impact of gender on FLE and LE. No significant gender differences were found in either foreign language enjoyment or learner engagement. The first half of the finding confirmed Jiang and Dewaele's (2019) research yet differed from other studies in which females reported higher levels of FLE than their male counterparts (Dewaele and MacIntyre, 2014; Dewaele et al., 2016, 2018). The latter half of the finding differed from previous research where female students were found to have a higher level of engagement (Oga-Baldwin and Nakata, 2017).

Thirdly, correlation analyses revealed a high positive correlation between participants' FLE and LE. In a larger picture, this echoes the prevalent conclusion supported by cognitive psychologists, social psychologists, and neuroscientists in laboratory studies that activating positive emotions, like the feeling of enjoyment, are critically important for students' engagement with academic tasks (Pekrun and Linnenbrink-Garcia, 2012). Again, this finding is consistent with recent research in SLA where FLE is found to facilitate or sustain learner engagement (e.g., Ryan and Patrick, 2001; Pekrun and Linnenbrink-Garcia, 2012; Reeve, 2012; Dincer et al., 2019; Mercer and Dörnyei, 2020). It further underpinned the broaden-and-build theory which argues that positive emotions like joy have the ability to broaden people's momentary thought-action repertoires (Fredrickson, 2001, 2013).

The aforementioned finding could further borrow support from the results of the multiple linear regression analyses which indicated that approximately 62% (Adjusted *R*^2^ = 0.614) of participant's engagement with their language learning could be explained by the feeling of enjoyment they experienced in EFL class. With respect to the subdomains of FLE, FLE-private was a stronger predictor of participants' engagement (*B* = 0.538) than FLE-social (*B* = 0.304). This result lends support to previous findings that EFL learners' self-concept, which was defined as “an individual's self-descriptions of competence and evaluative feelings about themselves as a FL learner” (Mercer, 2011, p. 14), was an important psychological antecedent of learner engagement (Mercer, 2019). Regression analyses further showed that the causal relationship between FLE and LE was reciprocal rather than unidirectional as the same proportion of FLE could be attributed to learner engagement. This finding echoes the results of Pekrun and Linnenbrink-Garcia's (2012) study that academic emotions were linked to their antecedents and effects by reciprocal causation over time. The high correlations between FLE and LE can be partly explained by the overlap of the two constructs in that feeling enjoyable, to some degree, means EFL learners are emotionally engaged in the class considering emotional engagement is one of the pivotal aspects of LE. More importantly, the interplay found between FLE and LE makes it safe to conjecture that FLE and LE are antecedents of each other. The actualization of FLE where EFL teachers have a big part to play will engage learners more and accordingly, learners' deep engagement in their study will boost their feeling of enjoyment.

Fourthly, the present study dealt with the interactions between FLE, LE and learners' academic achievements and absenteeism *via* correlation analysis. The results revealed that both FLE and LE had low, positive correlation with learner achievement. The positive correlation between participants' FLE and their achievements is consistent with similar findings in previous SLA studies (Pekrun et al., 2002; Pekrun and Linnenbrink-Garcia, 2012; Dewaele and MacIntyre, 2014; Piechurska-Kuciel, 2017; Dewaele and Alfawzan, 2018; Li et al., 2020a). The positive correlation between LE and achievement confirms the prevalent claim that engagement predicts achievement and attainment (Finn and Zimmer, 2012; Pekrun and Linnenbrink-Garcia, 2012). However, this low correlation was probably due to the fact that achievements in this study were not reported according to exact scores but on a 4-point scale, which might makes the differentiation a fuzzy area to explore. The results also revealed that a higher level of FLE-social was associated with a lower level of absenteeism. However, there was no significant correlation found between LE and absenteeism, which was not consistent with the original intention of engagement studies for dropout intervention (Reschly and Christenson, 2012).

Finally, the present study dealt with the sources of FLE and trends of LE. Results of the thematic analysis were consistent with the findings of the quantitative data. FLE was subject substantially to teacher-external variables including mainly stimulating classroom activities the teacher devised, positive classroom atmosphere the teacher directs, and harmonious teacher-student relationship the teacher dominate to create (Xie and Derakhshan, 2021). This finding lent support to previous studies (e.g., Dewaele and Dewaele, 2017, 2020; Dewaele et al., 2018; Jiang and Dewaele, 2019; Ahmadi-Azad et al., 2020). Besides, the statistical analysis also revealed that FLE-self was the second most important attactor of FLE. EFL learners' pursuit of new knowledge from class and, in the long run, utilitarian needs for good scores in multiple English proficiency tests were pivotal predictors of FLE. This finding was in line with Elahi Shirvan and Taherian's (2020) study where personal goals were found to be one of the two prototype contributors to FLE, the other being the teacher. However, FLE-peer was the least significant predictor of FLE in this study although peer support and friendship added to the feeling of enjoyment of some participants. This was probably due to the gap in students' English proficiency and people preferred to make progress through peer interaction rather than interacting for interaction's sake.

Thematic analysis of the trends of participants' LE was also consistent with the findings of the qualitative results. FLE was an important contributor of LE and Chinese EFL learners tended to engage themselves in English learning more emotionally, behaviorally and cognitively whereas they were not accustomed to the more active and aggressive agentic engagement owing to the uniqueness of the Chinese cultural and educational context discussed above.

## Conclusion

This present study conducted a sequential mixed-method study to examine the dynamics between Foreign Language Enjoyment (FLE) and learner engagement (LE), and their effects on EFL learners' academic achievement and absenteeism. It turned out that FLE was highly and positively correlated with LE and the causal relationship between them was reciprocal. To be more concrete, FLE-private had stronger power in predicting language learner engagement than FLE-social. In addition, both FLE and LE showed low correlations with participants' academic achievements and no significant correlation emerged between FLE or LE and absenteeism. Thematic analysis of the qualitative data further revealed that FLE was subject substantially to teacher-external variables such as stimulating classroom activities, positive classroom atmosphere, and teacher-student rapport. What is more, EFL learners' pursuit of new knowledge from class and utilitarian needs for good scores in multiple English proficiency tests made FLE-self the second most significant attractor of FLE. Analysis of the trends of LE indicated that Chinese EFL learners preferred to engage themselves in their English learning more emotionally, behaviorally and cognitively than agentically. Next, we suggest some pedagogical implications to enlighten the practice of EFL practitioners.

Several limitations of this present study should be noted. First, participants of this study were from three comprehensive universities in central China where university students had intermediate English proficiency level. This means the findings reported here might not predict the general trend of all Chinese undergraduate EFL learners. Future research could include participants with a broader range of language proficiency levels. Second, participants' achievements were reported on a much broader 4-point scale rather than according to exact scores. This might make the measurement of achievement less sensitive to the variation of foreign language enjoyment or learner engagement. Third, we could not find any correlation between learner engagement and participants' absenteeism despite that engagement studies initiated from dropout intervention (Reschly and Christenson, 2012). Last, there existed some extent of overlapping coding since some of participants' descriptions involved multiple themes that needed to be categorized differently.

Despite the limitations, the findings of this study have important pedagogical implications for EFL teaching in Chinese universities. To begin with, teachers should devise stimulating classroom activities with balanced challenges (Oxford, 2017; Dewaele et al., 2019; Mercer and Dörnyei, 2020), related to students' immediate concerns, allowing them autonomous chances to present themselves, enhancing interactions and encouraging positive atmosphere. Second, teachers should establish rapport in the classroom through being approachable, being supportive to students' learning enthusiasms, respecting students, and showing confirmation for them. Both the factors of teacher (with interpersonal treatments of immediacy, confirmation, care, and positive character traits) and positive classroom atmosphere with stimulating activities and encouraging interactions play an influential part in facilitating foreign language enjoyment (Mercer and Dörnyei, 2020; Xie and Derakhshan, 2021), which will, in turn, lead to desirable academic outcomes such as deep learner engagement and better achievement. Third, teachers should treat students as equals to establish a relaxing classroom environment instead of patronizing them as subordinates, and encourage them to exchange their preferences and opinions freely in order to boost the feeling of enjoyment and induce their deep engagement in language learning accordingly. Finally, FLE can be correlated with other teacher-student interpersonal variables (Xie and Derakhshan, 2021) and positive psychology variables (Wang et al., 2021) to investigate more effective ways to engage students in EFL learning and improve their academic attainment.

## Data Availability Statement

The raw data supporting the conclusions of this article will be made available by the author, without undue reservation.

## Ethics Statement

The studies involving human participants were reviewed and approved by Henan University Academic and Ethics Committee. The patients/participants provided their written informed consent to participate in this study.

## Author Contributions

The author confirms being the sole contributor of this work and has approved it for publication.

## Funding

This study was supported by the Humanities and Social Sciences Project of the Education Department of Henan Province of China under (Grant No. 2019-ZZJH-507) and the 13th Five-year Plan Project of Educational Science of Henan Province of China (No. 2020YB0034).

## Conflict of Interest

The author declares that the research was conducted in the absence of any commercial or financial relationships that could be construed as a potential conflict of interest.

## Publisher's Note

All claims expressed in this article are solely those of the authors and do not necessarily represent those of their affiliated organizations, or those of the publisher, the editors and the reviewers. Any product that may be evaluated in this article, or claim that may be made by its manufacturer, is not guaranteed or endorsed by the publisher.

## Appendix

### Semi-Structured Interview

1. Have you ever experienced enjoyment in your English class? If so, please describe the experience.
2. If one of your very close friends asks you what you think about this course and this teacher, what will you say?
3. Could you give details about your relationships with your teacher and classmates in the class?
4. How does being in this class make you feel about your English competency?
5. What kind of behaviors do you perform in the class to be successful?
6. Are you interested in classroom activities and the course? Why or why not? Please explain.
7. How do you feel in class?.
8. Do you do extra things that would help your learning when you are studying course-related concepts?
9. What kind of strategies do you follow when studying this course?
10. Do you ask questions that would help your learning in the class?
11. How do you express your opinions to your teacher in this course?
12. Could you give details about your course absenteeism and feelings when you do not attend the course?
13. How is your English achievement?
14. If you had a magical wand to change anything about this course, what would it be?
15. What factors make you enjoy learning English? (You can enumerate them based on their importance).
